# The role of Mediterranean diet in the epidemiology of metabolic syndrome; converting epidemiology to clinical practice

**DOI:** 10.1186/1476-511X-4-7

**Published:** 2005-04-12

**Authors:** Demosthenes B Panagiotakos, Evangelos Polychronopoulos

**Affiliations:** 1 Department of Nutrition and Dietetics, Harokopio University, Athens, Greece

**Keywords:** metabolic syndrome, diet, lifestyle modification, Mediterranean diet

## Abstract

Metabolic syndrome is a collection of associated conditions such as dyslipidemia, high blood pressure, impaired glucose tolerance and tendency to develop fat around the abdomen. It is now well known that individuals with the metabolic syndrome are at high risk for atherosclerosis and, especially, coronary heart disease. However, it has been suggested that people with the metabolic syndrome may benefit from aggressive lifestyle modification, through diet and exercise. In this review we summarize scientific evidence regarding the effect of Mediterranean diet on the development of metabolic syndrome.

## 

The metabolic syndrome is a collection of associated conditions such as dyslipidemia, high blood pressure, impaired glucose tolerance, and abdominal fat. It has first been described in 1988, and it is now widely adopted that it is a health situation that promotes atherosclerosis [1]. Each of the associated conditions has an independent effect, but clustering together they become synergistic, making the risk of developing atherosclerosis greater. Moreover, many investigators have shown a direct association between the prevalence of the syndrome with increased risk of cardiovascular disease and diabetes. Because each independent factor of the metabolic syndrome can increase the patient's cardiovascular risk, an integrated, comprehensive approach is indicated for patients with the syndrome. Treatment of metabolic syndrome is primarily based on Therapeutic Lifestyle Change, implementing weight-loss diets and exercise programmes to increase physical activity [2].

## What is the metabolic syndrome?

Definitions of the metabolic syndrome are confusing. Since 1999 several investigators and Organizations have suggested different definitions. Nevertheless, all agree that the characteristics of the metabolic syndrome include atherogenic dyslipidemia, a prothrombotic state, insulin resistance, hypertension, abdominal obesity, as well as elevated microalbuminuria, increased fibrinogen, decreased plasminogen activator, elevated plasminogen activator inhibitor-1, increased blood viscosity, and increased uric acid [1]. Each abnormality promotes atherosclerosis independently, but when clustered together, these metabolic disorders are increasingly atherogenic and enhance the risk of cardiovascular morbidity and mortality. Moreover, the metabolic syndrome has been associated with the development of stroke, type-2 diabetes, diabetic nephropathy, retinopathy, and distal neuropathy. In addition, the National Cholesterol Education Program Adult Treatment Panel (NCEP ATP) III [2] presents the metabolic syndrome as an enhancer of cardiovascular risk beyond elevated low-density lipoprotein cholesterol.

## Epidemiology of the Metabolic Syndrome

A substantial proportion of individuals living in Western nations are afflicted with multiple metabolic abnormalities. A recent report estimated that 115 million people in US, Japan, France, Germany, Italy, Spain and United Kingdom suffer from the metabolic syndrome [3]. Moreover, it is estimated that at least 47 million Americans have this condition, while by the year 2010 the number of US citizens that have this condition it is estimated to be between 50 to 75 million [1]. Ferrannini et al. [4] reported that more than 70% of adults have at least one of the major characteristics of the metabolic syndrome. The ATTICA Study investigators [5] recently reported that the prevalence of the metabolic syndrome was 25% in men and 15% in women from Greece. The prevalence of the metabolic syndrome in a south Mediterranean population (i.e. Greece) was similar with the prevalence of the syndrome in a sample of 8814 American men and women from the 3^rd ^National Health and Nutrition Examination Survey (1988–1994) (i.e. 22%) [1]. In a Korean population the prevalence of metabolic syndrome was 29.0% in men and 16.7% in women aged 30–80 years [6], while in another study of the same country the prevalence of the syndrome was 13% in both en and women [7]. Hu et al. [8] studying 6156 men and 5356 women without diabetes from 11 prospective European cohorts reported that the age-standardized prevalence of the metabolic syndrome was 15.7% in men and 14.2% in women. The prevalence of metabolic syndrome in a Portuguese sample was 27.0% in women and 19.1% in men [9]. Moreover almost all these studies observed that the prevalence of the syndrome increases with age. Differences in genetic background, dietary habits, levels of physical activity, population age and sex structure, levels of over- and under-nutrition, may influence the prevalence of both the metabolic syndrome and its components worldwide. Nevertheless, all these data suggest that the prevalence of the syndrome is high due to increasing obesity and sedentary lifestyles; and reflect the growing necessity for therapeutic intervention.

Recently the NCEP ATP III suggested for Therapeutic Lifestyle Changes [2] in order to reduce the prevalence of the metabolic syndrome. These changes included the consumption of low-saturated diet (<7% of total fat) and the adoption of a physically active lifestyle. In this review we focus our interest of the effect of diet and exercise on the prevalence of the metabolic syndrome. We particularly study the effect of a traditional diet, the Mediterranean diet, on the components of the syndrome, in relation to exercise.

## Diet and metabolic syndrome

Among several factors, related to lifestyle habits that could influence cardiovascular risk the beneficial effect of diet has already been underlined [10-12]. During the last decades there is increasing scientific evidence that there are protective health effects from diets, which are high in fruits, vegetables, legumes and whole grains and which include fish, nuts, and low-fat dairy products. Such diets need not to be restricted in total lipid intake fat as long as there is not an excess of energy intake over expenditure calories and emphasize predominantly vegetable oils that there are low in saturated fats and partially hydrogenated oils [13]. Foods are composed from six basic nutrients: (a) carbohydrates, (b) fat, (c) proteins, (d) vitamin, (e) minerals and (f) water. Its balance on daily dietary patterns has more or less related to the prevalence of many metabolic disorders, like hypertension, dyslipidemia, obesity and diabetes, as well as to increased risk of atherosclerotic disease.

The main purpose of carbohydrates is to provide energy for the body. Although the body can make energy from other sources, like fats and even proteins, the consumption of carbohydrates is special because it can provide energy without the use of oxygen, it is required for explosive types of work, it is the preferred energy for the brain, and the energy derived from carbohydrates is necessary to burn fats. However, carbohydrate consumption has been branded as the culprit in weight gains, obesity, diabetes, and a number of other diseases. Although carbohydrate consumption has been related to such health problems, it has been suggested that it is the excess consumption of the wrong carbohydrates that causes these health problems and not the proper carbohydrates consumed at proper amounts. Moreover, metabolic consequences of carbohydrates depend not only on their quantity but also on their quality. However, other people suggest that carbohydrates of any type may be bad for some people, particularly those with metabolic syndrome. The use of glycemic index as a measure of how fast a food raises your blood sugar is controversial [14]. Major health organizations discourage the use of the glycemic index in nutritional therapy, despite the fact that the concept has now been widely adopted by many international organizations. Although the evaluation of the glycemic index needs to be further improvements, especially within different populations, the concept of glycemic load is attractive because it captures both the quality and quantity of carbohydrates as well as potential interactions between them. Large amounts of complex carbohydrates, like such potatoes, breads, corn, etc. are recommended by several specialists. The Diet, Nutrition, and the Prevention of Chronic Diseases report of the World Health Organization recommend that 55% to 60% of the daily caloric intake should be obtained from carbohydrates. Forty-five to fifty percent of these calories should come from complex carbohydrates, and natural sugars found in fresh fruits and vegetables and no more than 10% from refined and processed sugars [13]. Nevertheless, the consumption of carbohydrates in people with the metabolic syndrome remains controversial and needs further investigation.

Fiber is an organic compound found in plants. It is found in the skin of fruits, seeds, leaves, stems and roots. High-fiber diets have received considerable attention in recent years, due to their connection with lower incidence of several metabolic disorders like blood pressure, diabetes, obesity, as well as heart disease and colon cancer.

Fat is a general term used that refers to oils, fats and waxes. There are two types of dietary fats: a) saturated and b) unsaturated. The unsaturated fats are further divided into the monounsaturated and polyunsaturated fats. Usually the daily energy intake consists 30% of fat, but no more than 10% of these calories should come from saturated (animal fats). The rest 20% should come from unsaturated (vegetable) oils. However, whatever the fat intake, certain oils must be included in the diet, like the essential fatty acids. They are polyunsaturated fats derived mostly from vegetable oils such as safflower oil, corn oil, olive oil and soybean oil. Lack of these oils in one's diet will cause series illness.

Proteins, like carbohydrates and fats, contain atoms of carbon, oxygen and hydrogen. In addition, proteins contain nitrogen. Proteins are made up of molecules called amino acids. These amino acids are strung together in a specific order and make a complete protein. Contrary to the beliefs of many, we only need relatively small amounts of protein for good health. The requirements for adults are 0.8 grams per kilogram of body weight.

Vitamins are organic compounds that are necessary for normal metabolism. They are manufactured in the green leaves and roots of plants, except vitamin B-12 that is mainly found in animal foods and products. It is a fact that vitamin deficiency leads to poor health and even death, while vitamin overdose can also lead to health problems. Thus, taking more vitamins than recommended will be of no benefit and may even be harmful. Vitamins like B-6 and B-12 have been associated with lower risk of cardiovascular disease, but this association is still under controversy.

Finally, minerals are metallic elements that play an important role in the metabolism and proper function of the body. Minerals occur freely in nature, and they are found in water, plants, and soil. Most of the major minerals (salt, potassium and iron) have been associated with some metabolic disorders and human health. Excessive consumption of salt causes hypertension in many people or aggravates existing hypertension. On the other hand potassium helps get rid of excess salt in the body. Foods reach in potassium includes bananas and all fruits, vegetables, beans and nuts (unsalted). Iron plays a key role in metabolism, performance and health. It is involved in the transport of oxygen to the tissues, and in a number of biochemical reactions responsible for energy production. Iron is absorbed by the body from iron found in the food consumed. Iron deficiency results in a reduction in the size of red blood cells, and the amount of hemoglobin they contain. The daily iron requirements for males are 10 mg, while females requires almost double. It is common essence that more or less all nutrient components are necessary for human health, however dietary habits should have a balanced intake of the aforementioned food components.

## The Mediterranean diet

Although different regions in the Mediterranean basin, have their own diets (like Spain, France, Italy, Greece, Cyprus, etc) it is legitimate to consider these as variants of a single entity, the Mediterranean diet [15]. During the past decades a large body of evidence related adherence to a Mediterranean diet with all causes mortality, prevalence of some metabolic disorders (like obesity, and high blood pressure), as well as incidence of coronary heart disease and various types of cancer [16-20].

This dietary pattern is mainly characterized by daily olive oil consumption. Olive oil is important not only just because it has several beneficial properties, but because it allows the consumption of large quantities of vegetables in the form of salads and equally large quantities of legumes in the form of cooked foods, too. Thus, it might be convenient, if not wholly accurate, defining Mediterranean diet as the dietary pattern found in the olive growing areas of the Mediterranean region, in the late 1950s and early 1960s, when the consequences of World War II were overcome, but the fast-food culture had not yet invaded the area. Other essential components of the Mediterranean diet are wheat, olives and grapes, and their various derivative products. Total lipid intake may be high, (around or in excess of 40% of total energy intake) in Greece, or moderate, (around 30% of total energy intake) in Italy. In all instances, however, the ratio of monounsaturated to saturated fats is much higher than in other places of the world, including northern Europe and North America. Specifically this dietary pattern could be described by the following components: (a) daily consumption: of non refined cereals and products (whole grain bread, pasta, brown rice, etc), vegetables (2–3 servings/day), fruits (4–6 servings/day), olive oil (as the main added lipid) and non-fat or low fat dairy products (1–2 servings/day), (b) weekly consumption: of potatoes (4–5 servings/week), fish (4–5 servings/week), olives, pulses, and nuts (>4 servings/week), and more rare poultry (1–3 servings/week), eggs and sweets (1–3 servings/week) and (c) monthly consumption: of red meat and meat products (4–5 servings/month) [15]. It is, also, characterized by moderate consumption of wine (1–2 wineglasses/day), mainly wine during the meals, and high monounsaturated-to-saturated fat ratio (>2). Additionally, although intake of milk is moderate, the consumption of cheese and yogurt is relatively high. Feta cheese is regularly added to salads and accompanies vegetable stews [15].

The relationship between Mediterranean diet and metabolic syndrome has been investigated in several studies. Several aspects of this dietary pattern have shown a beneficial effect on the development of the metabolic syndrome or its components. Particularly, fish and omega – 3 fatty acids intake, which are essential components of the Mediterranean diet, have been associated with a lower risk of cardiovascular disease. Kris – Etherton et al. in a recent review article summarize epidemiological studies and randomised clinical trials that showed a considerable of effect of fish consumption on cardiovascular system [21]. They concluded that eicosapentaenoic and docosahexaenoic acids supplementation ranging from 0.5 to 1.8 gr per day (either as fatty fish or supplements) significantly reduces subsequent cardiac and all-cause mortality. The mechanisms are not fully understood, but there are strong evidences to support that omega-3 fatty acids intake are associated with lower levels of blood pressure, triglycerides, reduced endothelial activation, and other factors associated with the metabolic syndrome [21]. Moderate alcohol consumption, has been associated with low prevalence of the metabolic syndrome. In particular Rosell et al. [22], in a cross-sectional survey of about 4200 middle aged men and women from Stockholm county in Sweden, observed that the metabolic syndrome was significantly more common in non-drinkers (20%), and less common among wine drinkers (8%), compared to a group with low alcohol intake. Moreover, after various adjustments made the investigators observed 40% lower odds ratio for the metabolic syndrome only in women who consumed wine. Many researchers related Mediterranean diet with improvements in blood pressure and lipid profile (especially LDL cholesterol and triglycerides), decreased risk of thrombosis (i.e. fibrinogen levels), improvement in endothelial function, and insulin resistance, reduction in plasma homocysteine concentrations and decrease in ventricular irritability [23-27]. Particularly, Esposito et al. [24] in a randomized clinical trial among men and women with the metabolic syndrome instructed 90 patients in the intervention group to follow a Mediterranean-style diet (i.e. crease daily consumption of whole grains, fruits, vegetables, nuts, and olive oil), while patients in the control group to follow a prudent diet (carbohydrates, 50%–60%; proteins, 15%–20%; total fat, <30%). After two years, patients consuming the Mediterranean diet had significantly reduced serum concentrations of C-reactive protein, interleukin – 6, insulin resistance, as well as improved endothelial function score. Recently, Psaltopoulou et al. [26] from the Greek cohort of the European Prospective Investigation into Cancer and Nutrition (EPIC) study that included about 20000 healthy people from all regions of the country, observed that greater adherence to the Mediterranean diet was significantly inversely associated with both systolic and diastolic blood pressure. Particularly, intakes of olive oil, vegetables, and fruit were inversely associated with both systolic and diastolic blood pressure, whereas cereals, meat and meat products, were positively associated with arterial blood pressure. Furthermore, antioxidants represent a common element in the Mediterranean pattern and an antioxidant action provides a plausible explanation for the apparent benefits. In a recent review article, Vissers et al. [28] searched the MEDLINE database for the years 1966–2002 and observed that phenol-rich olive oil lowers oxidisability of ex vivo low-density lipoprotein particles or lowers markers in urine of oxidative processes in the body. However, these effects of phenols were not found in human studies. It is known that wild edible greens frequently eaten in the form of salads and pies contain very high quantities of flavonoids. Although there is no direct evidence that these antioxidants are central to the benefits of the Mediterranean diet, indirect evidence from epidemiological data and the increasing understanding of their mechanisms of action suggest that antioxidants may play a major role. Finally, adoption of the Mediterranean diet has been associated with reduced inflammation process, too. In particular, Chrysohoou et al. studied 1514 men and 1528 women from the ATTICA study and observed that greater adherence to the Mediterranean diet (upper tertile of the diet score) was associated with 20% lower C-reactive protein, 17% lower interleukin-6, 15% lower homocysteine, 14% lower white blood cell and 6% lower fibrinogen, as compared to those who were in the lowest tertile, after various adjustments were made [29]. All these findings provided scientific evidences regarding the pathways that relate diet and cardiovascular diseases.

A potential explanation of the beneficial effect of this dietary pattern on human health is because it is low in saturated fat, high in monounsaturated fat, mainly from olive oil, high in complex carbohydrates, from legumes, and high in fibre, mostly from vegetables and fruits. The high content of vegetables, fresh fruits, cereals and olive oil, guarantee a high intake of b-carotene, vitamins C and E, polyphenols and various important minerals. These key elements have been suggested to be responsible for the beneficial effect of diet on human health, and especially cardiovascular disease.

In nutritional epidemiology, interest has shifted from the study of single nutrients or foods, to the study of food groups and, more recently, dietary patterns. The single-nutrient approach fails to account for the interactions between nutrients and does not take into consideration that some nutrients are correlated between them. In this context, the study of dietary patterns, and that of the Mediterranean dietary pattern, has considerable interest. Recently, based on a cross sectional survey in an urban area in Greece (the ATTICA Study), we showed that adherence to this dietary pattern has been associated with 20% lower odds of having the metabolic syndrome, irrespective of age, sex, physical activity status, lipids and blood pressure levels [5]. Williams et al. [30] supported the hypothesis that dietary patterns rich in salads, vegetables, fruits, fish, pasta and rice and low intake in monounsaturated fats are associated with glucose intolerance and various other features of the metabolic syndrome. In particular, he studied 802 middle aged subjects that underwent an oral glucose-tolerance test, and observed that a healthy balanced diet close to the Mediterranean diet was associated with reduced central obesity, fasting plasma glucose, 120 min non-esterified fatty acid and triacylglycerol, and positively correlated with HDL-cholesterol. These results provided further evidence for the recommendation of a healthy balanced diet as one of the main components of chronic disease prevention. The CARDIO2000 study investigators studying 848 patients with a first event of an acute coronary syndrome and 1078 people without any evidence of cardiovascular disease, from all Greek areas, focused their interest in the relationship between Mediterranean diet and cardiovascular risk in people with the metabolic syndrome. They observed that the adoption of the Mediterranean diet was associated with a 35% reduction of the coronary risk in subjects with the metabolic syndrome, after adjusting for various potential confounders [31]. More recently, Esposito et al. [24] showed that a Mediterranean-style diet seems effective in reducing the prevalence of the metabolic syndrome and its associated cardiovascular risk. Particularly, at the end of the follow up 44% patients in the intervention group still had features of the metabolic syndrome, compared to 87% patients in the control group. This may lead to a 51% risk reduction of having the syndrome due to this dietary intervention.

## Conclusion

Dietary approaches to treating and preventing metabolic syndrome vary, but nearly all experts agree that clinical parameters are greatly improved through various dietary changes and body weight control. Whether adoption of Mediterranean diet is a recipe for the metabolic syndrome seems to have a scientific basis; however, it needs further investigation by randomised clinical trials. Nevertheless, health care professionals need to help people understand the benefits from the introduced dietary patterns and support them to adopt these lifestyle characteristics.

## References

[B1] Hansen BC (1999). The metabolic syndrome X. Ann NY Acad Sci.

[B2] (2001). Executive Summary of the Third Report of the National Cholesterol Education Program (NCEP) Expert Panel on Detection, Evaluation, and Treatment of High Blood Cholesterol in Adults (Adult Treatment Panel III). JAMA.

[B3] Ford ES, Giles WH, Dietz WH (2002). Prevalence of the metabolic syndrome among US adults: findings from the third National Health and Nutrition Examination Survey. JAMA.

[B4] Ferrannini E, Natali A (1991). Essential hypertension, metabolic disorders, and insulin resistance. Am Heart J.

[B5] Panagiotakos DB, Pitsavos CH, Chrysohoou C, Skoumas J, Tousoulis D, Toutouza M, Toutouzas PK, Stefanadis C (2004). The Impact of Lifestyle Habits on the Prevalence of the Metabolic Syndrome among Greek adults from the ATTICA study. Am Heart J.

[B6] Oh JY, Hong YS, Sung YA, Barrett-Connor E (2004). Prevalence and factor analysis of metabolic syndrome in an urban Korean population. Diabetes Care.

[B7] Lee WY, Park JS, Noh SY, Rhee EJ, Kim SW, Zimmet PZ (2004). Prevalence of the metabolic syndrome among 40,698 Korean metropolitan subjects. Diabetes Res Clin Pract.

[B8] Hu G, Qiao Q, Tuomilehto J, Balkau B, Borch-Johnsen K, Pyorala K, DECODE Study Group (2004). Prevalence of the metabolic syndrome and its relation to all-cause and cardiovascular mortality in non-diabetic European men and women. Arch Intern Med.

[B9] Santos AC, Lopes C, Barros H (2004). Prevalence of metabolic syndrome in the city of Porto. Rev Port Cardiol.

[B10] Kannel WB, McGee DL, Gordon T (1976). A general cardiovascular risk profile: The Framingham Study. Am J Cardiol.

[B11] Appel L, Moore T, Obarzanek E, Vollmer W, Svetkey L, Sacks F, Bray G, Vogt T, for the DASH Collaborative Research Group (1997). A Clinical Trial of the Effects of Dietary Patterns on Blood Pressure. N Engl J Med.

[B12] Wood D (2001). Established and emerging cardiovascular risk factors. Am Heart J.

[B13] World Health Organization Study Group (2003). Diet, Nutrition, and the Prevention of Chronic Diseases. Geneva, Switzerland: World Health Organization; Technical Report Series, 916,.

[B14] Schulze MB, Hu FB (2004). Dietary approaches to prevent the metabolic syndrome. Diabetes Care.

[B15] Trichopoulou A., Lagiou P (1997). Healthy traditional Mediterranean diet – An expression of culture, history and lifestyle. Nutr Rev.

[B16] Trichopoulou A, Lagiou P, Kuper H, Trichopoulos D (2000). Cancer and Mediterranean dietary traditions. Cancer Epidemiol Biomarkers Prev.

[B17] Kris-Etherton P, Eckel R, Howard B, Jeor S, Bazzare T (2001). Lyon Diet Heart Study. Benefits of a Mediterranean-Style, National Education Program/AHA Step I Dietary Pattern on Cardiovascular Disease. Circulation.

[B18] Panagiotakos DB, Pitsavos C, Chrysohoou C, Tzioumis K, Papaioannou I, Stefanadis C, Toutouzas PK (2002). The Association of Mediterranean Diet With Lower Risk of Acute Coronary Syndromes, in Hypertensive Subjects. Int J Cardiol.

[B19] Trichopoulou A, Costacou T, Bamia C, Trichopoulos D (2003). Adherence to a Mediterranean diet and survival in a Greek population. N Engl J Med.

[B20] Martinez-Gonzalez MA, Fernandez-Jarne E, Serrano-Martinez M (2002). Mediterranean diet and reduction in the risk of a first acute myocardial infarction: an operational healthy dietary score. Eur J Nutr.

[B21] Kris-Etherton P, Harris W, Appel L (2002). Fish Consumption, Fish Oil, Omega-3 Fatty Acids, and Cardiovascular Disease. Circulation.

[B22] Rosell M, De Faire U, Hellenius ML (2003). Low prevalence of the metabolic syndrome in wine drinkers – is it the alcohol beverage or the lifestyle?. Eur J Clin Nutr.

[B23] Knapp HW (1997). Dietary fatty acids in human thrombosis and hemostasis. Am J Clin Nutr.

[B24] Esposito K, Marfella R, Ciotola M, Di Palo C, Giugliano F, Giugliano G, D'Armiento M, D'Andrea F, Giugliano D (2004). Effect of a Mediterranean-style diet on endothelial dysfunction and markers of vascular inflammation in the metabolic syndrome: a randomized trial. JAMA.

[B25] Ruit-Gutierrez V, Muriana FJG, Guerrero A (1996). Plasma lipids, erythrocyte membrane lipids and blood pressure of hypertensive women after ingestion of dietary oleic acid from two different sources. J Hypertens.

[B26] Psaltopoulou T, Naska A, Orfanos P, Trichopoulos D, Mountokalakis T, Trichopoulou A (2004). Olive oil, the Mediterranean diet, and arterial blood pressure: the Greek European Prospective Investigation into Cancer and Nutrition (EPIC) study. Am J Clin Nutr.

[B27] Assmann G, de Basker G, Bagnara S (1997). International Consensus statement on olive oil and the Mediterranean diet: implications for health in Europe. Eur JCancer Prev.

[B28] Vissers MN, Zock PL, Katan MB (2004). Bioavailability and antioxidant effects of olive oil phenols in humans: a review. Eur J Clin Nutr.

[B29] Chrysohoou C, Panagiotakos DB, Pitsavos C, Das UN, Stefanadis C (2004). Adherence to the Mediterranean diet attenuates inflammation and coagulation process, in healthy adults: the ATTICA study. J Am Col Cardiol.

[B30] Williams DE, Prevost AT, Whichelow MJ, Cox BD, Day NE, Wareham NJ (2000). A cross-sectional study of dietary patterns with glucose intolerance and other features of the metabolic syndrome. Br J Nutr.

[B31] Panagiotakos DB, Pitsavos C, Kokkinos P, Chrysohoou C, Vavuranakis M, Stefanadis C, Toutouzas P (2003). The Adoption of Mediterranean Diet Attenuates the Development of Acute Coronary Syndromes in People with the Metabolic Syndrome. Nutr J.

